# Spectrometry of the Earth using Neutrino Oscillations

**DOI:** 10.1038/srep15225

**Published:** 2015-10-22

**Authors:** C. Rott, A. Taketa, D. Bose

**Affiliations:** 1 Department of Physics, Sungkyunkwan University, Suwon 440-746, Korea; 2 Earthquake Research Institute, University of Tokyo, 1-1-1 Yayoi, Bunkyo-ku, Tokyo, Japan

## Abstract

The unknown constituents of the interior of our home planet have provoked the human imagination and driven scientific exploration. We herein demonstrate that large neutrino detectors could be used in the near future to significantly improve our understanding of the Earth’s inner chemical composition. Neutrinos, which are naturally produced in the atmosphere, traverse the Earth and undergo oscillations that depend on the Earth’s electron density. The Earth’s chemical composition can be determined by combining observations from large neutrino detectors with seismic measurements of the Earth’s matter density. We present a method that will allow us to perform a measurement that can distinguish between composition models of the outer core. We show that the next-generation large-volume neutrino detectors can provide sufficient sensitivity to reject extreme cases of outer core composition. In the future, dedicated instruments could be capable of distinguishing between specific Earth composition models and thereby reshape our understanding of the inner Earth in previously unimagined ways.

Understanding the inner structure and composition of the Earth is fundamental to Earth science. While Earth’s matter density distribution can be inferred from geophysical observations, its compositional structure is far more difficult to determine. The state and composition of the core, which constitutes 32% of Earth’s mass and 16% of its volume, remains largely uncertain. The core consists of iron-nickel mixture and is divided into inner and outer regions distinguished by a large density difference at a depth of approximately 5,100 km. The inner core is solid, while the lack of s-wave propagation in the outer core and lower density indicate it to be liquid. The density deficit in the outer core, however, cannot be simply explained by a difference in state, but rather requires the presence of light elements at about 5 wt% (weight percent) to 10 wt%. There is great excitement in Earth science with regard to determining these light components in the outer core in order to understand the evolution of the Earth and the geodynamo. We introduce a new technique based on neutrino oscillations in order to remotely measure electron density and demonstrate how, in the near future, this method could be used to distinguish between different composition models of the inner Earth.

Analyses of seismic waves have resulted in the well-understood shell structure of the Earth, consisting of crust, upper mantle, lower mantle, outer core, and inner core. The matter density structure of the Earth has been accurately determined by combining astronomic-geodetic parameters, free oscillation frequencies, and seismic wave velocity measurements^1^. The composition of the crust near the surface can be measured directly. Drill core samples have resulted in composition measurements down to a depth of approximately 12 km^2^. The upper mantle composition can be probed through eruption entrainment sampling^3^. The state and composition of the Earth’s core, at a depth of approximately 2,900 km remains far more uncertain with no prospects of sampling materials.

The outer core composition can be inferred to be mostly iron-nickel mixture with traces of light elements, by combining seismological velocity profiles and the composition of primitive meteorites^4^. Through recent progress in high-pressure experiments, hydrogen, carbon, oxygen, silicon, and sulfur have been suggested as light element candidates^5^. However, the abundance of these light elements remains uncertain.

Obtaining reliable estimates for the abundances of light elements in the Earth’s core is essential to understanding the formation and evolution of the Earth^6^ and to determining the origin of the geomagnetic field^7^, which are two of the major problems in Earth science.

Neutrinos (denoted *ν*) are remarkable particles that have enjoyed an ever more important role in particle physics, cosmology, and astrophysics since they were predicted by theorist Wolfgang Pauli in 1930 and first observed in 1956^8^. There exist three different types (referred to as flavours) of neutrinos, *ν*_*e*_, *ν*_*μ*_, and *ν*_*τ*_, which relate to how the neutrino was produced. However, a neutrino’s flavour can change. For example, a neutrino produced as a *ν*_*μ*_ can be detected as a *ν*_*e*_. This process, which solved the solar neutrino problem^9^, is known as neutrino oscillation^10^. At a later date, three flavour neutrino oscillation was confirmed by observing the neutrinos produced in the Earth atmosphere^11^. Neutrino oscillations are a quantum mechanical consequence of neutrinos having mass, and as such the behaviour of these oscillations can be described precisely.

In the present study, we propose a novel technique for measuring the average chemical composition of the deep Earth using neutrinos. Due to their tiny interaction cross section, neutrinos can pass through the entire Earth without interacting. As mentioned earlier, due to neutrino oscillations, a flavour of one neutrino can convert to another flavour. Neutrino oscillations depend on the medium traversed, or, more specifically, on the electron density along the path of the neutrino through the Earth^12^. The compositional structure of the Earth can be obtained as the average ratio of the atomic number to the atomic weight (Z/A), by comparing the electron density distribution and the Earth’s matter density distribution. This effect makes neutrinos unique messenger particles to remotely probe the Earth’s interior.

Large-volume neutrino detectors have emerged as powerful tools in particle physics and astrophysics. Operating instruments have demonstrated their tremendous potential in groundbreaking discoveries, such as the observation of high-energy extra-terrestrial neutrinos by IceCube and through the observation of neutrino oscillations by Super-Kamiokande. There is a great interest in constructing the next generation of neutrino detectors with larger volumes and improved performance. This new generation of large-volume detectors could be capable of observing neutrinos at sufficiently high rates to perform the first experimental measurement of the Earth’s interior. With the advent of Hyper-Kamiokande (Hyper-K)^13^ and the Precision IceCube Next-Generation Upgrade (PINGU)^14^, spectrometry using neutrino oscillations could be demonstrated and a first direct measurement of the compositional structure of the Earth performed. Even more visionary ideas, such as large ocean-going^15^ or ice-based detectors, could see neutrino spectrometry emerge as a precision science capable of distinguishing different compositional models.

Preceding research of geophysics using neutrinos can be divided into three categories: (1) measurement of the radioactive nuclei density in the Earth using geo-neutrinos generated through nuclear decays, (2) measurement of Earth’s matter density using neutrino absorption, and (3) measurement of Earth’s matter density using neutrino oscillations^16^,^17^,^18^,^19^,^20^,^21^,^22^,^23^. In the present study, we introduce a new fourth category. We apply neutrino oscillations for a composition measurement, exploiting the fact that neutrino oscillations are dependent on electron density, which is the product of the matter density and the ratio of the average atomic number to the atomic weight. While the underlying physical phenomena are well understood, we focus in particular on the relevance of these effects to geophysics and discuss the prospects for an Earth composition measurement that could be performed within the next two decades.

## Results

### Neutrino oscillations in the Earth

In geophysics, neutrinos have received attention due to the information on the inner Earth they provide, as demonstrated by the measurement of radiogenic heat generated in the Earth through the observations of neutrinos from nuclear decays of uranium and thorium^24^,^25^. The success in detecting these geoneutrinos has confirmed the feasibility of using neutrinos in Earth science. While geoneutrinos are generated through nuclear decay and carry energies of approximately 10^6^ eV (one electron volt (eV) = 1.602 × 10^−19^ joules), the neutrinos used for the proposed method have energies of a few GeV (10^9^ eV) and are naturally produced when energetic cosmic rays collide with the upper Earth’s atmosphere.

**Figure 1:**
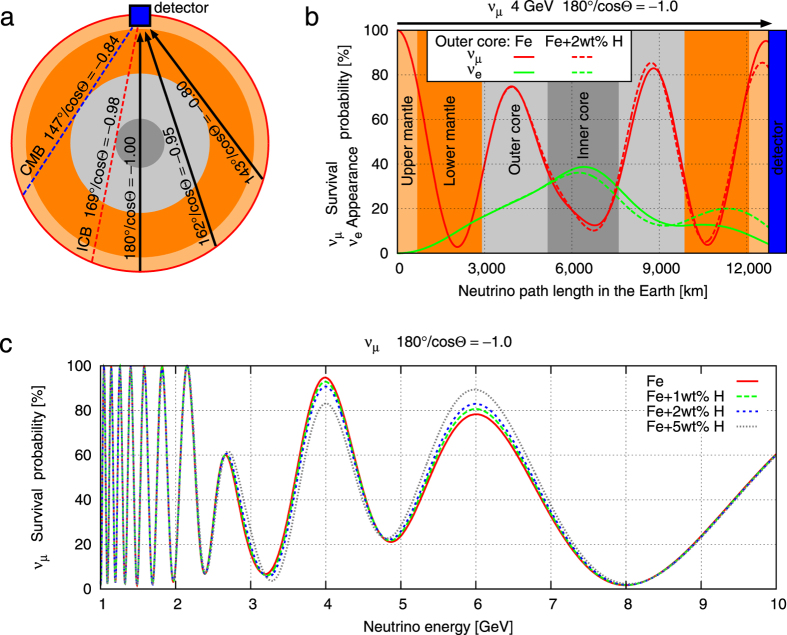
(**a**) Schematic diagram of a neutrino’s path through the Earth and the corresponding zenith angles. The inner core boundary (ICB) at 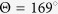 and the core mantle boundary (CMB) at 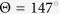 are indicated by dashed red and blue lines, respectively. (**b**) *ν*_*e*_ appearance probability (green) and *ν*_*μ*_ survival probability (red) as functions of path length in the Earth. The neutrino direction is 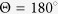, as shown in (**a**). The solid/dashed line corresponds to the case in which the composition of the outer core is pure iron/a mixture of iron and 2 wt% hydrogen. (**c**) 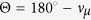 survival probabilities as a function of neutrino energy for different outer core compositions. The solid (red), long dashed (green), short dashed (blue), and dotted (gray) lines represent iron, a mixture of iron and 1 wt% hydrogen, a mixture of iron and 2 wt% hydrogen, and a mixture of iron and 5 wt% hydrogen, respectively.

The majority of atmospheric neutrinos produced are type *ν*_*μ*_, and their flavour changes as they pass straight through the Earth. The neutrino oscillation probability depends on a set of oscillation parameters, the neutrino energy, *E*_*v*_, the distance travelled, and the electron density along its path. The path length, L, is the distance that the neutrino travels from its point of origin in the atmosphere to the detector. Since all neutrinos relevant for this analysis are generated in the Earth’s atmosphere, L is simply a function of the zenith angle, Θ, of the neutrino arrival direction at the detector. Figure 1(a) shows the neutrino path through the Earth.

We calculate neutrino oscillation probabilities, following the approach of Barger *et al.*^12^ and use the numerical implementation of the NuCraft software package^26^. The oscillation parameters, which are well measured, are taken from the global fit given by Capozzi *et al.*^27^, assuming the case of a normal mass hierarchy. We use the modified Preliminary Reference Earth Model (PREM) matter density model^1^,^28^ to describe the Earth density and structure. We fix the mantle composition to pyrolite, the hypothetical mixture of distinct minerals, which accurately represent the constituents of the Earth’s mantle^4^. We constrain the composition of the inner core to iron and only vary that of the of the outer core. Figure 1(b) shows the *ν*_*μ*_ survival probability and the *ν*_*e*_ appearance probability as a function of the path length for a neutrino with an energy of 4 GeV (10^9^ eV) passing vertically through the Earth. The survival probability is the probability that a created neutrino of specific flavour is observed as such. In this case, we consider a muon neutrino observed as such 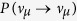. The appearance probability is the chance that a neutrino of one flavour is observed as a neutrino of a different flavour, for example 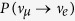. The flavour change as a function of the distance travelled in the Earth is shown. In order to visually show the impact of the outer core composition on the oscillation probability, we compare the cases of a mixture of iron and 2 wt% hydrogen with iron. Figure 1(c) shows the *ν*_*μ*_ survival probability at the surface of the Earth, as a function of the neutrino’s energy for four different core compositions. In order to visualize the difference in survival probability for different outer core compositions, we selected (1) iron, (2) a mixture of iron and 1 wt% hydrogen, (3) a mixture of iron and 2 wt% hydrogen, and (4) a mixture of iron and 5 wt% hydrogen as extreme examples of the outer core composition.

### Z/A ratios for different outer core models

Iron is the most abundant element in the outer Earth core and throughout this document we have chosen pure iron as our default composition. Models adding single or multiple elements to iron have been proposed^29^,^30^,^31^. In Table 1, we introduce some selected outer core composition models and characterize them according to Z/A ratio. The estimated maximal abundance of light elements^5^,^32^ for mixtures of iron are listed in Table 1. Note that nickel is thought to co-exist with iron in the outer core, with an estimated content of approximately 5%^33^. Since there is only a slight difference between Z/A values, using an mixture of iron and 5 wt% nickel as the base composition rather than iron will result in only a marginal change in Z/A from 0.4656 to 0.4661.

### Oscillation probabilities for different outer core models

**Figure 2:**
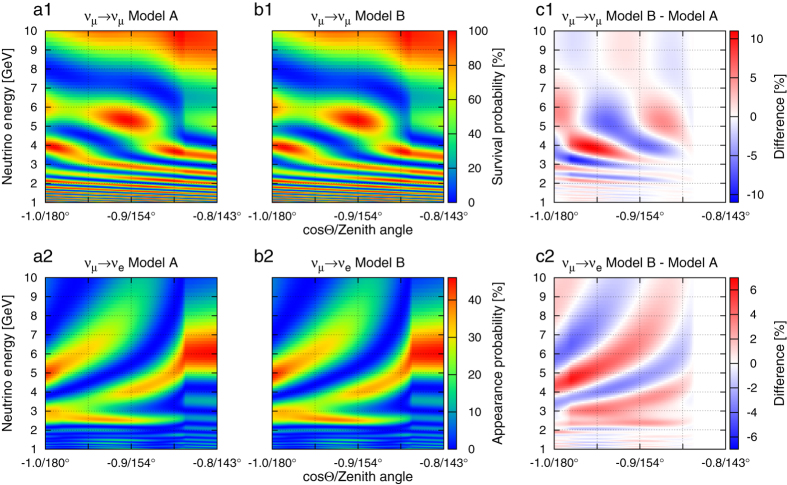
Comparison of oscillation probabilities for two different core compositions: Model A – iron; Model B – an mixture of iron and 2 wt% hydrogen. a1 and b1 show the *ν*_*μ*_ survival probabilities as a function of neutrino energy and zenith angle for Models A and B, respectively. a2 and b2 show the appearance probability for *ν*_*μ*_ to *ν*_*e*_ for Models A and B, respectively. c1 shows the difference between a1 and b1, and c2 shows the difference between a2 and b2.

As neutrino oscillations simply depend on the neutrino’s energy, path length, and composition along the path, we can determine the probability that a neutrino will change flavours as a function of the zenith angle and the energy. We calculated the oscillation probabilities for different core models. Figure 2 shows such an oscillogram, i.e., oscillation probabilities as a function of zenith angle and neutrino energy, for two different outer core compositions. Subtle differences in the neutrino survival probability can be exploited in order to distinguish between different composition models. The most pronounced differences in survival probability are for neutrinos with energies between 2 GeV and 8 GeV that traverse the outer core, i.e., their zenith angles are larger than 147°.

### Detector requirements

The described differences in neutrino oscillation effects that depend on the Earth’s composition could be detectable with a neutrino detector if the detector combines good energy and angular resolution in the relevant energy range and observes GeV neutrinos at sufficiently high rates to accumulate sufficient statistical samples. Due to the small neutrino interaction cross section, a large detector volume of megaton scale is necessary in order to acquire a sufficient number of neutrino events and not suffer from limited statistics. Good neutrino flavour identification can be beneficial.

### Neutrino detectors

Neutrinos can easily be detected through Cherenkov light emissions from one or more energetic particles produced in neutrino interactions in an optical transparent medium such as ice or water. The IceCube neutrino telescope^34^ is the world’s largest neutrino detector. It uses one gigaton (1,000 megatons) of ice at the Geographic South Pole that was instrumented with more than 5,000 photosensors. The optical sensor array relies on the ultra-pure Antarctic ice as a detection medium, in this way the giant detector size was realized in a cost-effective manner. The detector is working extremely well, and the recent discovery of high-energy astrophysical neutrinos demonstrates the potential of large neutrino detectors^35^. Precision physics with neutrinos of a few GeV has been pioneered by Super-Kamiokande^36^. This neutrino detector consists of a 50 kiloton water tank surrounded by 11,000 photosensors to observe Cherenkov light, allowing neutrino energies to be determined precisely and neutrino flavours to be identified reliably. The underlying technology applied by IceCube and Super-Kamiokande is well established and is the basis for future detectors. Next-generation detectors could benefit from better photosensors with higher photon detection efficiency.

### Sensitivity of benchmark detectors

We calculate the significance with which the composition of the outer core could be determined for some benchmark neutrino detectors. We use a generic neutrino detector description based on performance parameters to estimate sensitivities. Our parameterization can easily be converted into hardware and design requirements for the planning of new detectors. We compute expected event rates as a function of the neutrino energy and the zenith angle as a function of the product of detector size and the exposure time in megaton-years. In this way, we calculate the number of neutrino events for a certain energy, direction, and flavour. In our calculations we treat neutrinos (*ν*) and their anti-particles, the anti-neutrinos 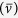, separately. As we refrain from discussing detectors with separation capabilities between *ν* and 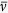, we use in the following the term neutrino for both *ν* and 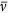, unless other specified. We create templates of the expected event rates for different outer core models. Event rates were calculated from the atmospheric neutrino flux, oscillation probabilities, neutrino cross section, detector volume, and exposure time. The atmospheric neutrino flux and energy spectrum are well understood for our purposes. We adopt the atmospheric neutrino flux model of Athar *et al.*^37^. For the neutrino (*ν*_*μ*_) and anti-neutrino 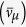 interaction cross sections, we use the approximate values of 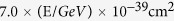 and 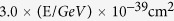, respectively^38^.

**Figure 3:**
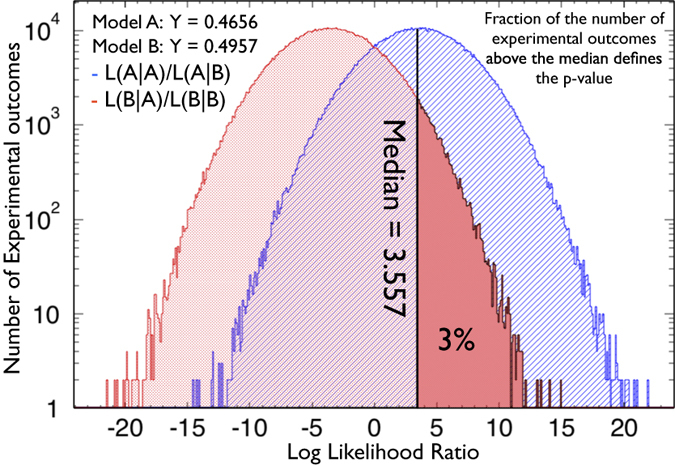
Method to determine significance of the expected experimental outcome using pseudo-experiments. The significance is defined as the fraction of outcomes in which the log of the likelihood ratio is above the median of the distribution with interchanged models. For the example shown here it can be seen that Model A (Iron) can be distinguished from Model B (Pyrolite) with a 97% confidence. We assumed an exposure of 10 MTyrs and the benchmark detector with energy resolution and angular resolution, as defined by 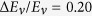 and 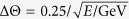.

The outcome of any experimental measurement that deals with individual events, such as the detection of neutrinos, will be subject to statistical fluctuations. We consider a large number of potential experimental outcomes, called pseudo experiments, in order to estimate the chance that models could be distinguished through an actual measurement. For each pseudo experiment, we compare the number of observed events to the number of expected events for a specific model. We calculate events for given ranges of energy and zenith angle. For each of these bins in energy and zenith angle, events follow Poisson statistics and we determine the probability for the observation. We then compute the likelihood of this experimental outcome with respect to a specific Earth model assumption. The total likelihood is then given by the product of the likelihoods for the individual bins. In order to compare the likelihood of one model with that of another, we compute the likelihood ratio. For the calculation of the expected significance, we perform a set of pseudo experiments and apply the log-likelihood ratio (LLR) method^39^. Figure 3 illustrates the significance calculation.

For simplicity of the analysis, we consider only muon neutrino 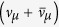 events, which are the most relevant for neutrino spectrometry. Muon neutrino events can be identified with high efficiency by proposed next-generation detectors, such as PINGU or Hyper-K. For PINGU, the resolution for the muon neutrino energy is expected to be better than 25% at 5 GeV, and the zenith angle resolution for the neutrino has been reported to be approximately 13°^14^. At Hyper-K, better angular and energy resolutions compared to PINGU can be expected, in addition to a larger than 99% efficiency to identify the interaction products of a neutrino interaction^13^,^40^.

**Figure 4:**
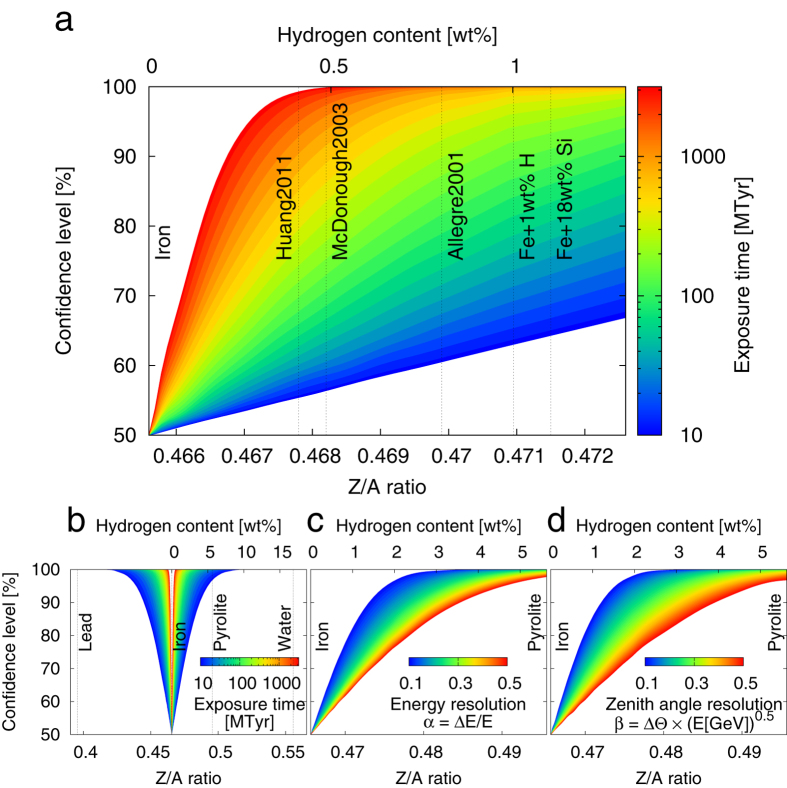
(**a**) Expected confidence level for rejecting a specific outer core composition with respect to iron plotted as a function of the corresponding Z/A ratio. A generic detector case with an energy resolution of 20% and an angular resolution of 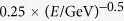 is shown as an example. The colour indicates the exposure time given in megaton-years. We indicate the Z/A ratios for some selected outer core composition models (see Table 1 for details) as black dotted vertical lines. (**b**) The same plot as (**a**) for a larger Z/A range. Sensitivity dependences on (**c**) energy resolution and (**d**) angular resolution for a generic detector with an exposure time of 30 megaton-years for an angular resolution of 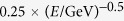 and an energy resolution of 20%, respectively.

Figure 4(a,b) show the sensitivity for rejecting outer core compositions given by their Z/A ratios with respect to iron for a generic neutrino detector. The detector is characterized by energy resolution and angular resolution, as defined by 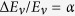 and 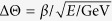, respectively. We choose *α* = 0.20 and *β* = 0.25 as a default generic benchmark detector and show the sensitivity depending on the product of lifetime and detector volume (megaton-years) in Fig. 4(b). The specified benchmark detector parameters correspond to a 20% energy resolution independent of energy and a 6.5° zenith angle resolution at 5GeV. The angular and energy resolution dependences of the sensitivity is shown in Fig. 4(c,d).

With an acquired dataset of 10 megaton-years, a neutrino detector could for the first time confirm an iron-like core through experimental measurements. Extreme cases of outer core composition such as lead or water could be rejected with more than 99% confidence with respect to iron. The Z/A ratios of iron, lead, mantle (pyrolite), and water are 0.4656, 0.3958, 0.4957, and 0.5556, respectively. The large hydrogen content appearing at the axis at the top of each plot could be rejected.

Figure 4(a) shows the future prospects of neutrino spectrometry. A one-gigaton-year (or 1,000 megaton-year) dataset, which is equivalent to observation for 20 year using a 50 megaton detector, would provide the ability to discriminate between established outer core composition models. Furthermore, the hydrogen content of the outer core could be measured with a precision of 0.4 wt%. Better sensitivities could be achieved if the detector exceeds the benchmark detector performance parameters selected for use in the present study as shown in Fig. 4(c,d) for the energy resolution and angular resolution, respectively.

Note that neutrino spectrometry determines the Z/A ratio. Based on this measurement Earth composition models can be distinguished by their corresponding Z/A ratios. Some Earth composition models however predict very similar values for this ratio. The origin for this lies in the fact that for example oxygen, sulfur, silicon, and carbon are being nearly degenerate in their ratio of atomic number to atomic mass. Hence, a model with Fe (90 wt%) + O (10 wt%) (Z/A = 0.4690) could be distinguished from an iron (Z/A = 0.4656), but would be indistinguishable from a model with Fe (90 wt%) + S (10 wt%) (Z/A = 0.4689) through neutrino spectrometry alone. The full impact of neutrino spectrometry can only be assessed by its complimentarily to existing methods in Earth science. For example, degeneracies between composition models could be resolved by using neutrino spectrometry in the combination with high-pressure experiments. High-pressure experiments measure the matter properties, such as density and sound velocity, of mixtures of iron with other light elements at core pressure-temperature conditions^41^. Measurements have resulted in limits on the content of each of the light elements in the core. Further studies, which are beyond the scope of this work, will be needed to show how a combination of neutrino spectrometry with high-pressure experiments could distinguish composition models.

## Uncertainties

We discuss the feasibility of the neutrino spectrometry measurement with future neutrino detectors with respect to theoretical and experimental uncertainties.

Uncertainties in the composition measurement originate from limited knowledge of the neutrino oscillation parameters, atmospheric neutrino flux uncertainties, neutrino cross section uncertainties, and uncertainties in the Earth’s matter density profile. In addition to these theoretical uncertainties, detector related uncertainties must be determined. However, this is beyond the scope of the present study and would have to be carried out through experimental collaborations.

**Figure 5:**
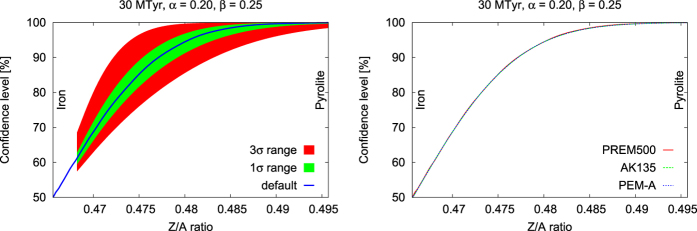
Systematic error of the expected confidence level as a function of Z/A. Left: Systematic error resulting from the uncertainty in oscillation parameters. The blue line represents default mixing parameter case, the red area represents its 3*σ* uncertainty range, and the green area represents its 1*σ* uncertainty range. A generic detector case with an exposure time of 30 MTyr, an energy resolution of 20%, and an angular resolution of 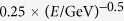 is shown. Right: Systematic error resulting from matter density models. Expected confidence level for rejecting a specific outer core composition with respect to iron plotted as a function of the corresponding Z/A ratio. A generic detector case with an exposure time of 30 MTyr, an energy resolution of 20%, and an angular resolution of 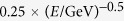 is shown. We estimated the confidence level using three different density models. The solid (red), dashed (green), and dotted (blue) lines represent the modified PREM, AK135, and PEM-A, respectively.

To examine the feasibility of the neutrino spectrometry measurement, we calculated the confidence level by changing the oscillation parameters and the matter density models. To calculate the confidence level, we used the same oscillation parameters and matter density model for each template. Using the current best-fit oscillation parameters and their uncertainty^27^, the error in the confidence level curve was approximately ±4% at 90% (see Fig. 5:Left). To calculate the uncertainty from oscillation parameters, we performed a grid scan of the oscillation parameters, each of them was varied with a 0.1*σ* step size for the 1*σ* uncertainty range, and a 0.25*σ* step size for the 3*σ* range. Several experiments are planned for the near future in order to realize more precise measurements of the neutrino mixing parameters^42^,^43^,^44^. A better determination of the neutrino oscillation parameters will reduce uncertainties.

In order to estimate the uncertainty resulting from the matter density models, we have calculated the confidence level curves using three different density models (PREM500^1^,^28^, AK135^45^, PEM-A^46^; see Supplementary Figure 1). The systematic error resulting from the matter density in the confidence level curve was negligible compared with the systematic error resulting from the mixing parameters (see Fig. 5:Right). The expected uncertainty is sufficiently small to distinguish the models introduced in the present study. However, in order to determine the light material contents in the outer core, a more precise mixing parameter and matter density model, which may be available in the near future, are needed. We have limited our sensitivity study to the dominant systematic uncertainties. Capozzi *et al.*^47^ showed that systematic uncertainties related to resolution functions in energy and angle are sub-dominant to the oscillation parameter uncertainties. We note that effects related to the detector energy and angular resolution deserve further investigations, but go beyond the scope of this work.

At present, the neutrino mass hierarchy, one of the fundamental neutrino properties, remains unknown^27^,^48^. By the time that the proposed composition measurement is performed, we can safely assume that the mass hierarchy will have been determined, potentially even at the same neutrino detector considered for our measurement^49^,^50^. For the case of a normal (inverted) neutrino mass hierarchy matter induced neutrino oscillations will act on neutrinos (anti-neutrinos). A normal mass hierarchy would be favourable for our proposed measurement because the atmospheric flux of neutrinos is larger than that of anti-neutrinos and the interaction cross section of neutrinos is larger compared to that of anti-neutrinos. For our study we have assumed a normal mass hierarchy. In the case of an inverted mass hierarchy a roughly six times larger dataset would be needed to achieve the same sensitivity compared to the normal mass hierarchy.

## Discussions

Neutrino oscillations provide a way to distinguish different Earth composition models by probing the Z/A ratio. The novel method introduced here can provide a measurement of the chemical abundances of the inner Earth, which is direct, independent, and complimentary to phase state measurements in high pressure experiments. A better measurement of the core composition is essential to improve scenarios of the formation of the Earth, which depend on composition models^6^. Further, a composition measurement is essential to understand convection in the outer liquid core. Understanding the convection effects is key to explain the gyodynamo, which is expected to be driven by thermal and compositional convection in the outer core^7^. Neutrino spectroscopy will lead us to a better understanding of the Earth’s evolution and the origin of the geomagnetic field.

Large-volume neutrino detectors with good angular and energy resolutions at neutrino energies of 2−8 GeV are needed for our proposed measurements. While at present no instrument exists that combines the necessary capabilities discussed here, the technology is however well established and proven through large volume and high-precision detectors. In the near future however, new neutrino telescopes and upgrades to existing instruments will significantly enhance neutrino detection capabilities in the most relevant energy range for the spectroscopic measurement discussed here. The Hyper-K project will see the construction of a 0.6-megaton fiducial volume detector comprising eight compartments and approximately 100,000 photosensors. PINGU will use a few megatons of clearest ice in the centre of the IceCube detector. A detector of similar size named as ORCA is also considered as a deep-sea neutrino telescope in the Mediterranean Sea as part of the KM3NeT project^51^,^52^. Construction of a gigaton volume detector (GVD)^53^ has also started in lake Baikal and complementation with a PINGU-like detector could be considered. All these detectors in the planning and construction phase offer sufficient sensitivity to demonstrate neutrino spectrometry measurements and are expected to exclude extreme cases of the Earth’s composition, such as water or lead. If complimented and carefully optimized for neutrino spectrometry, the first meaningful bounds on the hydrogen content in the core could be in reach. In the future, dedicated next-generation neutrino experiments could be used to distinguish between different composition models, as we have demonstrated.

We have limited our studies to muon neutrinos. The detection of neutrinos of different flavours is expected to enhance the sensitivity of the proposed method, warranting further investigation in the future.

In the present study, we focused on the composition of the outer core, but neutrino spectrometry could also be applied to the mantle, especially in order to elucidate the water content of the lower mantle. Through recent progress in diamond inclusion sampling, high-pressure experiments, and dense seismic velocity measurements, it was found that the uppermost part of the lower mantle can reserve 1 wt% water^54^. Neutrino spectrometry has the ability to provide an upper limit for the water content of the lower mantle in the same way as the hydrogen content of the outer core.

## Methods

### Flux calculation

Event distributions for a generic neutrino detector defined by volume, energy resolution, and angular resolution were calculated for angle-averaged atmospheric neutrino fluxes after propagation through the Earth. The calculation proceeded in two steps: (1) first we calculated the transition probabilities for neutrinos of all flavours as function of zenith angle and energy. For this step we computed full three-flavour neutrino oscillations with the NuCraft software package^26^ using the 400 layers PREM Earth structural model. The results were binned in 720 bins of the cosine of the zenith angle and 400 bins of energy to obtain a transfer matrix *T*. We computed *T* for different outer core Z/A ratios and for the purpose of systematic studies, we used different Earth structural models (see Supplementary Figure 1) and oscillation parameters. (2) In a second step we obtain detector event rates. The predicted neutrino flux for muon neutrinos obtained from the Honda model^37^,^55^ was propagated through the Earth using transition tables describing the muon neutrino survival probability. Interaction rates were calculate for our generic detector and coarsely binned in 40 bins of the log of reconstructed neutrino energy and in 20 bins of the cosine of the reconstructed zenith angle for the range of 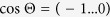 and log(E/GeV) = 0…1. The transitions to this coarser binned matrix *M* was done to have bin sizes that are closer to expected detector resolutions and to insure a reasonable event statistics per bin, avoiding empty bins. To obtain reconstructed zenith angle and neutrino energies we randomly sampled from the expected distribution as defined by the detector model (assuming Gaussian distributions). For each bin of *T*, 100,000 events were generated and mapped into the reconstruction matrix, weighted by the expected neutrino flux calculation^37^. Rates were calculated according to the neutrino interaction cross section and the product of the detector volume and operation time (megaton-years). Each bin *m*_*ij*_ of *M* obtains the expectation value of an observation, were a measurement to be performed. Using a different Earth composition model and the same generic detector model, we can obtain the expectation values for this model. Let 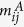 and 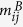 be the templates for two different models, respectively A and B.

### Log-likelihood method

Ensembles of pseudo datasets are drawn from each template 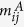 and 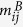 that are repeatedly varied following Poisson statistics. The log of the Poisson likelihood of the pseudo data for a specific bin is calculated with respect to the corresponding bin in 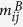 and 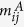. Given an experimental outcome *O*, with bins *o*_*ij*_, we take the sum of 
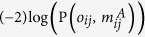
 to obtain the total likelihood. In this way, for each pseudo dataset, two likelihoods are calculated and are labelled 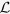 (pseudo data, template). The likelihoods are used to calculate LLR. We calculate two distributions for pseudo data *o*_*ij*_ drawn from 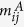 given by 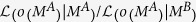 and 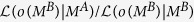. A total of 10,000 pseudo datasets are used to achieve adequate coverage of the probability space. We obtain expected significances from our ensemble of pseudo experiments. The probability of distinguishing model *A* from *B* is obtained by calculating the fraction of cases in which events drawn from *M*^*A*^ have a likelihood ratio that is more consistent with *M*^*A*^ than *M*^*B*^. An illustration of this method is given in Fig. 3.

## Additional Information

**How to cite this article**: Rott, C. *et al.* Spectrometry of the Earth using Neutrino Oscillations. *Sci. Rep.* **5**, 15225; doi: 10.1038/srep15225 (2015).

## Supplementary Material

Supplementary Information

## Figures and Tables

**Table 1:** Z/A ratios for mixtures of iron and light elements and some selected composition models.

**Model name**	**Z/A ratio**	**O(wt%)**	**C(wt%)**	**S(wt%)**	**H(wt%)**	**Si(wt%)**
Single-light-element model (maximum abundance)						
Fe + 11 wt%O^32^,^56^	0.4693	11	—	—	—	—
Fe + 12 wt%C^5^	0.4697	—	12	—	—	—
Fe + 13 wt%S^5^	0.4699	—	—	13	—	—
Fe + 1 wt%H^5^	0.4709	—	—	—	1	—
Fe + 18 wt%Si^32^	0.4715	—	—	—	—	18
Multiple-light-element model						
Huang2011^31^	0.4678	0.1	—	5.7	—	—
McDonough2003^30^	0.4682	0	0.2	1.9	0.06	6
Allegre2001^29^	0.4699	5	—	1.21	—	7
