# Supplementary material for: Spectrometry of the Earth using Neutrino Oscillations
Source: Sci Rep. 2015 Oct 22;5:15225. doi: 10.1038/srep15225 (PMC4614908; doi:10.1038/srep15225)
Supplement: Supplementary Information [file srep15225-s1.pdf]

Supplementary Materials

Spectrometry of the Earth using Neutrino Oscillations

C. Rott[1], A. Taketa[2], D. Bose[1]

[1] *Department of Physics, Sungkyunkwan University, Suwon 440-746, Korea.*

[2] *Earthquake Research Institute, University of Tokyo, 1-1-1 Yayoi, Bunkyo-ku, Tokyo, Japan.*

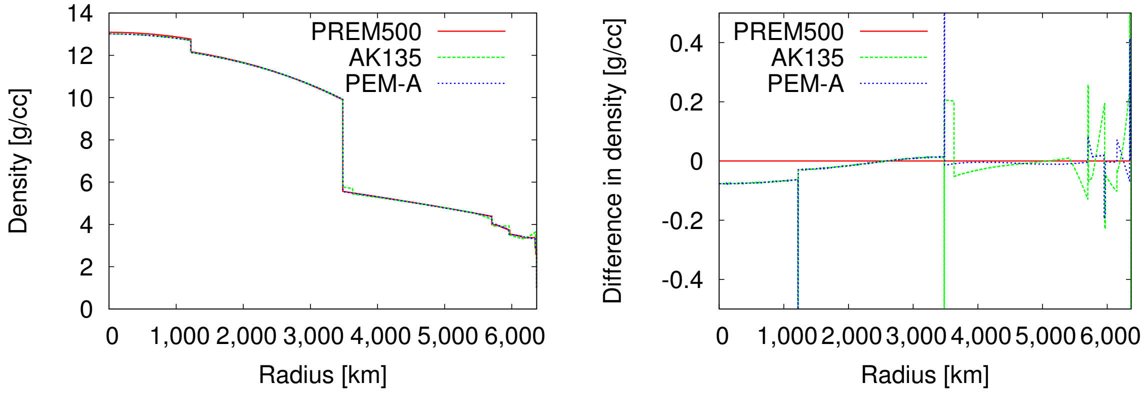

Supplementary Figure 1: left: Matter density distributions of the Earth as a function of radius from the centre of the Earth. The solid (red), dashed (green), and dotted (blue) lines represent the modified PREM, AK135, and PEM-A, respectively. right: Difference from modified PREM (PREM500).
